# Comparative evaluation of orthodontic bracket base shapes on shear bond strength and adhesive remnant index: An *in vitro* study

**DOI:** 10.4317/jced.53869

**Published:** 2017-07-01

**Authors:** Dennis Pham, Prashanti Bollu, Kishore Chaudhry, Karthikeyan Subramani

**Affiliations:** 1 Roseman University of Health Sciences, College of Dental Medicine, Henderson, NV, USA

## Abstract

**Background:**

The objective of this study was to evaluate the effect of orthodontic bracket base shape on shear bond strength and adhesive remnant index.

**Material and Methods:**

In this *in vitro* study using 140 bovine incisors, shear bond strength (SBS) of brackets with different base shapes (rectangle, flower, round, heart, diamond, star, and football) were measured with an Instron testing machine and tested until bond failure. Adhesive Remnant Index (ARI) scoring was evaluated after debonding to evaluate the location of bond failure. Descriptive and one-way ANOVA post-hoc Tukey statistical analyses were performed with a statistical significance set at *p*≤0.05.

**Results:**

Statistically significant difference in mean SBS in Newtons was observed for multiple base shapes (*p*<0.05). The highest mean SBS (N ± SD) was observed in football and flower base shapes (73.83 N ± 53.46; 65.82 N ± 37.89 respectively); the lowest mean was observed with diamond and heart shapes (30.51 N ± 11.73; 33.28 N ± 16.89 respectively). When reported in Megaspascals, statistically significant difference was observed for rectangle base shape (3.54 MPa ± 2.69) when compared to all other base shapes.

**Conclusions:**

Bracket base shape has an effect on SBS. Higher SBS (N) for rectangle, flower, and football base shape indicates even stress distributions throughout the bracket base. Base shape with a pronounced converging tip over the axial plane may contribute to the reduction in SBS due to increased peak stress concentration resulting in bond failure.

** Key words:**Shaped brackets, Shear bond strength, Adhesive remnant index, WildSmiles®.

## Introduction

Presently, there is a growing desire towards superior aesthetic appliance during orthodontic treatment (1-3). Patients undergoing treatment today can choose from wide array of aesthetic options including smaller stainless steel braces, clear braces with tooth color matching properties and lingual braces. However, these products are designed to meet the demand of aesthetically conscious patient population comprised mostly of adults. Typical population under orthodontic care consist mainly of children and adolescents between the ages of 9-14 (4,5). Since they are estimated to make up over 50% of orthodontic patient population, development of orthodontic appliances that are attractive to these age groups is necessary. Not until recently has there been a study, which investigated orthodontic appliance preference for children and adolescents. Study performed by Walton *et al.* concluded that children and adolescents are less concerned with aesthetics during treatment but instead prefer an orthodontic appliance that is unique (5). Specifically, the most preferred fixed orthodontic appliance within these age groups was shaped orthodontic bracket (5).

The introduction of shaped orthodontic bracket has been relatively new. WildSmiles® (Omaha, NE, USA) is credited with the development of this unique and innovative product. Shaped bracket encompasses similar components as the traditional bracket, the difference being the incorporation of unique base shape as follows: flower, soccer (round), heart, diamond, star, and football. Since the bracket base directly attaches to the enamel surface, the effect of this modification on tooth adherence needs to be investigated. Ideally, an orthodontic bracket must be able to withstand normal masticatory forces without being dislodged (6). Maximum occlusal force for children between the ages of 6-11 and adults with normal facial height is approximately 5.01 Kg and 13.5 Kg respectively (6). Clinically acceptable shear bond strength (SBS) within the range of 5.8-7.9 MPa has also been suggested to be ideal (7). Values below this range may increase the risk of bracket failure during treatment and values above this range increases the risk of enamel surface damage during debonding.

Several studies have investigated various factors within the bracket base component and its effect on bond strength (6,8-11). These factors include bracket base surface area, mesh wire gauge, number of mesh layers and retention base designs. MacColl *et al.* investigated the effect of bracket base surface area on SBS by comparing different base sizes (6). He concluded there were no differences in SBS for surface area within the range of 6.82-12.35 mm2 (6). However, significant reduction in SBS was observed if the surface area was below 6.82 mm2. The effect of the mesh wire size was also performed. Cucu *et al.* investigated the effect of mesh wire gauge by comparing 80-gauge versus 100-gauge mesh (8). Sharma *et al.* also performed a similar study but included a 60-gauge wire size in addition to the 80, and 100-gauge mesh (9). In both studies, no difference in SBS was found between an 80-gauge vs 100-gauge. Sharma *et al.* sought the effect of mesh layer on SBS (9). They concluded that the number of mesh layer was not a determining factor as there was no difference in SBS observed. Bishara *et al.* also conducted a similar study by comparing SBS of bracket that utilized a single 81-gauge versus a double 81-gauge mesh layer (10). The findings from this study coincided with the result from previous study. Lastly, Wang *et al.* examined the influence of retention base design on SBS (11). The retention base designs evaluated are as follows: retention groove, circular concave, single mesh and double mesh layers with varying size. This study concluded that retention base design of a bracket could affect SBS.

Due to its recent introduction of shaped orthodontic bracket, there are no published studies investigating the effect of bracket base shape on bond strength. Evaluating the effect of modification made to this vital component that plays a significant role in tooth adherence is necessary. In addition, the effect of bracket base shape on SBS has not yet been determined. The objective of this study was to evaluate the effect of orthodontic bracket base shape on SBS.

## Material and Methods

-Test Samples

In this *in vitro* bond strength study, SBS in Newtons (N) and in Megapascals (MPa) of seven orthodontic bracket base shapes were measured with an Instron testing machine (Instron Model E1000, Boston, MA) and compared. The control group consisted of an orthodontic bracket with a traditional rectangular base shape (American Orthodontics® Master Series 0.022” slot MBT prescription). The test groups were comprised of shaped brackets (WildSmiles® 0.022” slot Twin MBT prescription) with six different base shapes; flower, soccer (round), heart, diamond, star, and football. 140 maxillary central incisor orthodontic brackets (n=20/shape) were used in this study for all shapes due to its minimal curvature within the bracket base.

-Specimen Selection

Due to the need for non-carious, sound tooth structure in large quantities, bovine incisors were used as a substrate in this study. The bovine incisors were obtained from Animal Technologies® (Tyler, TX). 142 out of 160 bovine incisors met the selection criteria as follows.

• Minimal facial contour

• No gross damage on the enamel surface (under 10x magnifications)

- Gross damage was defined as any surface defect < 0.5 mm in depth or in width

-Specimen Mounting and Preparation 

Each bovine incisor was mounted on a base prior to Instron testing using ¾” diameter x 2” PVC coupler (Dura Plastic Products Inc., CA). Incisor root was embedded within the 1” layer of play sand (Pavestone Co., NV). A 2”x 4” plastic jig helped achieve proper tooth orientation, ensuring that bonding surface was flat and perpendicular to the base. Two-part epoxy (Devcon®, MA) was mixed and poured into the PVC base, surrounding the bovine root completely and set to cure. The mounted specimen was stored in an artificial saliva solution (Biotene®,GlaxoSmithKline, NJ) to prevent desiccation.

-Bracket Bonding

Bonding surface of bovine incisor was polished for 15 seconds with non-fluoride containing pumice using low-speed handpiece with a dental prophylaxis cup attachment. The enamel surface was then etched using 35% phosphoric acid gel (Opal Etch, Ultradent®, UT) for 15 seconds and rinsed thoroughly. It was air-dried using an air-water syringe until chalky appearance on the treated surface was observed. Adhesive primer (Opal Seal, Ultradent®, UT) was applied to the etched enamel surface with a microbrush then light cured for 10 seconds. Orthodontic bracket adhesive (Opal Bond MV, Ultradent®, UT) was applied and manipulated into the bracket mesh base with a plastic spatula. The bracket was pressed onto the enamel surface with an approximate force of 300 grams, measured with a Dontrix force gauge. Residual cement around the periphery of the bracket base was removed using an orthodontic scaler. From a standardized distance of 6mm, the orthodontic bracket was light cured for 10 seconds from the mesial, distal, incisal and gingival aspects, for a total duration of 40 seconds. The prepared samples were stored in an artificial saliva solution (Biotene®) until machine testing.

-Instron Testing

**Figure 1:**
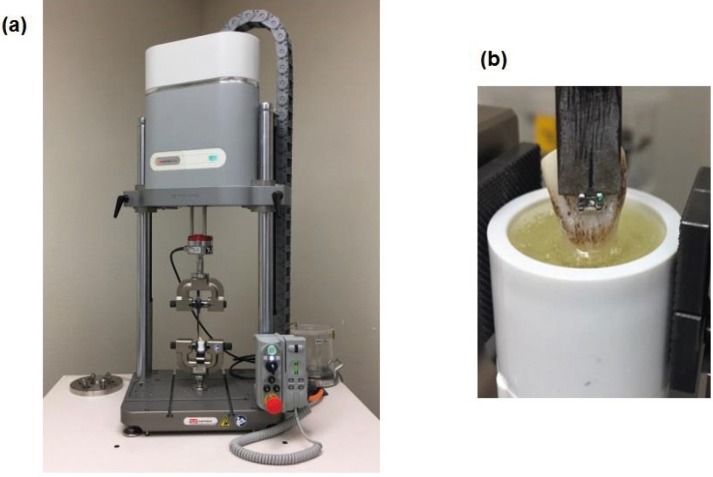
(a) Instron testing set up with the specimen held in position. (b) Instron attachment blade placed at the bracket ligature groove applying a static incisal-gingival force at a crosshead speed of 1mm/min.

Shear bond test was performed using the Instron testing machine. A blade placed at the bracket ligature groove (Fig. 1) applied a static incisal-gingival force at a crosshead speed of 1mm/min. Due to various geometrical base shapes, blade placement at this location maintained consistent force application throughout all test groups. SBS in Newtons (N) at bracket failure was recorded for each sample. SBS in MPa was then calculated by dividing the bracket failure force (N) by its respective nominal base area in square millimeters.

-Adhesive Remnant Index (ARI) 

Qualitative analysis using ARI score post Instron testing was performed under 10 x magnification to determine the location of bond failure. Due to its inherent subjectivity associated when evaluating ARI score, this process was performed twice at two-week interval to ensure reliability. The breakdown of ARI score is as follows (12).

ARI score:

0 = no adhesive remaining on enamel

1 = less than 50% adhesive remaining on enamel

2 = more than 50% adhesive remaining on enamel

3 = all adhesive remaining on enamel

-Statistical Analysis

Statistical analyses were performed using IBM SPSS Statistics Version 23.0 (IBM, Armonk, NY). Descriptive statistics including mean SBS (N), SBS (MPa), and ARI score with standard deviation was recorded. One-way analysis of variance (ANOVA) and post-hoc Tukey, with significance level set at 0.05 helped determine the presence of statistical difference between all groups.

## Results

**Figure 2:**
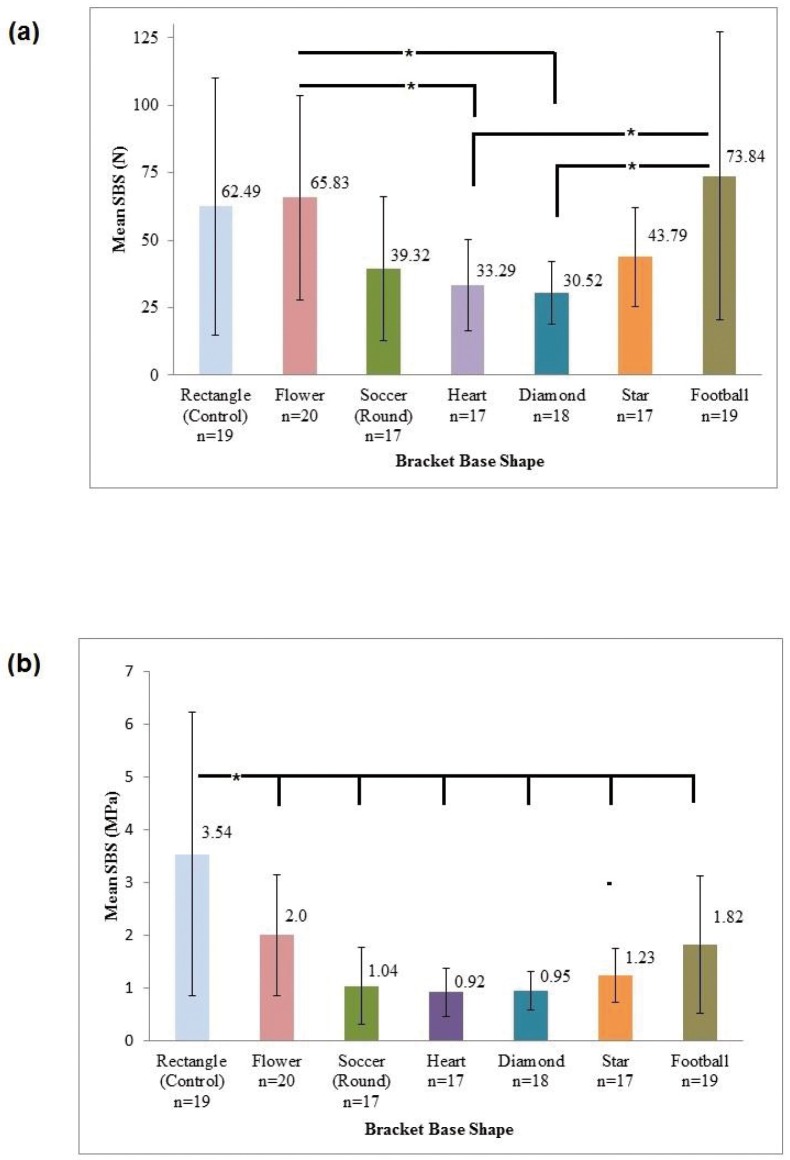
(a) Mean Shear Bond Strength of Brackets with different base shapes in Newtons (N). The results are shown in Mean ± SD. Overall ANOVA *p*-value <0.05. Post-hoc Tukey: Control group was not statistically significant with other groups (*p* >0.05). Statistically significant between-group comparison (*p*<0.05): Flower/Diamond, Flower/Heart, Football/Diamond, and Football/Heart base shape. (b) Mean Shear Bond Strength of brackets with different base shapes in Megapascals (MPa). The results are shown in Mean ± SD. Overall ANOVA *p*-value <0.05. Post-hoc Tukey: Control group was statistically significant with all test groups (*p*<0.050.01).

Samples where the brackets were sheared off at force level of N<10 were omitted as it represented total bracket failure. The mean SBS (N) with standard deviation (Mean ± S.D) with respect to different bracket base shapes is shown in figure 2a, which showed overall ANOVA *p*-value to be <0.05. Post-hoc Tukey inter-group comparison revealed no statistical differences in mean SBS (N) between control (62.49 N ± 47.57) and all other test groups. However, inter-group comparison between experimental groups revealed statistically significant difference in mean SBS (N) between flower/diamond, flower/heart, football/diamond, and football/heart base shapes (*p*<0.05). The highest mean SBS (N) observed was football and flower base shape (73.83 N ± 53.46 and 65.82 N ± 37.89 respectively) whereas the lowest mean SBS (N) was observed with diamond and heart shape (30.51 N ± 11.73 and 33.28 N ± 16.89 respectively).

Similar analyses were also conducted for SBS (MPa). The results from descriptive and one-way ANOVA statistical analyses for all base shapes are shown in figure 2b, which also showed statistically significant differences (*p*<0.05). Post-hoc Tukey multi-group comparison revealed statistically significant difference in mean SBS (MPa) between control and all other test groups (*p*<0.01). No statistical difference was observed for all other groups. No statistical difference was observed for all other groups.

Frequency distribution with mean ARI score (Mean ± S.D.) is shown in  Table 1. One-way ANOVA post-hoc Tukey group comparison revealed statistically significant differences for diamond (1.28 ± 0.83) and football (1.26 ± 0.89) when compared against star (1.94 ± 0.24) base shape (*p*<0.05) (Overall ANOVA *p*-value <0.05.

**Table 1:**
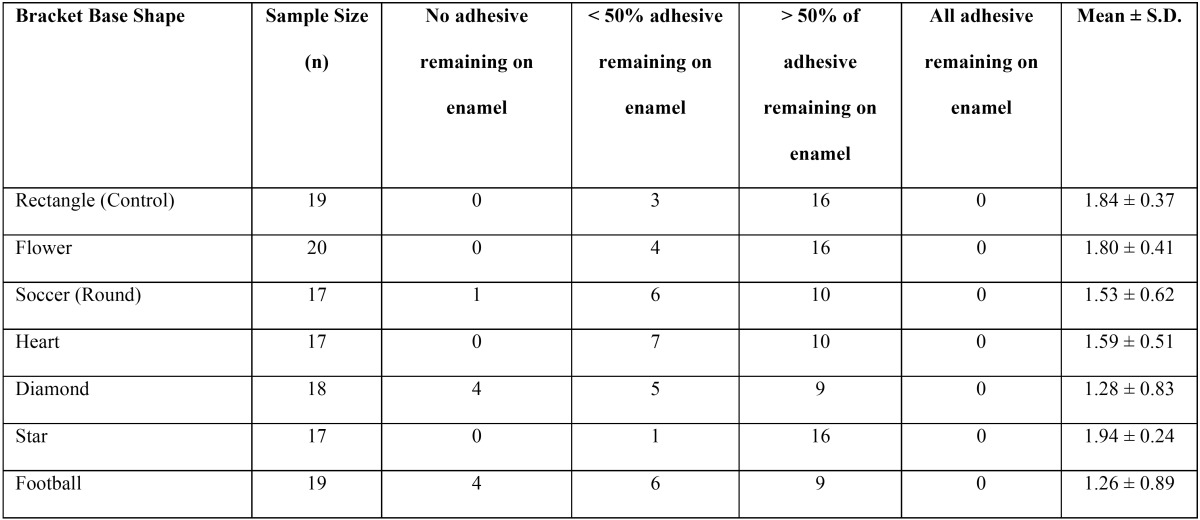
Adhesive Remnant Index Frequency Distribution Table with Mean Score ± SD.

## Discussion

Rectangular base shape yielded the highest, statistically significant SBS (MPa) when compared against all other base shapes: flower, round, heart, diamond, star and football. However, football and flower yielded a higher total SBS (N) than rectangle base shape. The contributory cause of these contradictory results is potentially due to discrepancy in base size. Since MPa is calculated as a function of its respective nominal base surface area (mm2), different bond values are achieved for different bonding area (13). Despite the previous claim of linear correlation between increased base size and ability of bracket to withstand dislodging forces, this relationship may only be accurate for a typical standard bracket with base surface area in the range of 6.82 – 12.35 mm2 (6,14). The nominal base surface area for the rectangular base shape used in this study was 17.63 mm2; all other base shapes exhibited a significantly larger base surface area within the range of 32.26 – 40.58 mm2. These significantly larger bases were uncommon until the recent introduction of shaped brackets; hence, it would be reasonable to conclude that different base size-bond strength relationship may exist above a particular surface area. Although the total SBS (N) for rectangle base shape was similar to football and flower, its smaller base resulted in significantly higher SBS (MPa) value after conversion. Alternately stated, lower SBS MPa value for both football and flower base shapes indicate force dissipation over greater surface area, resulting in the reduction of total force per unit area.

**Figure 3:**
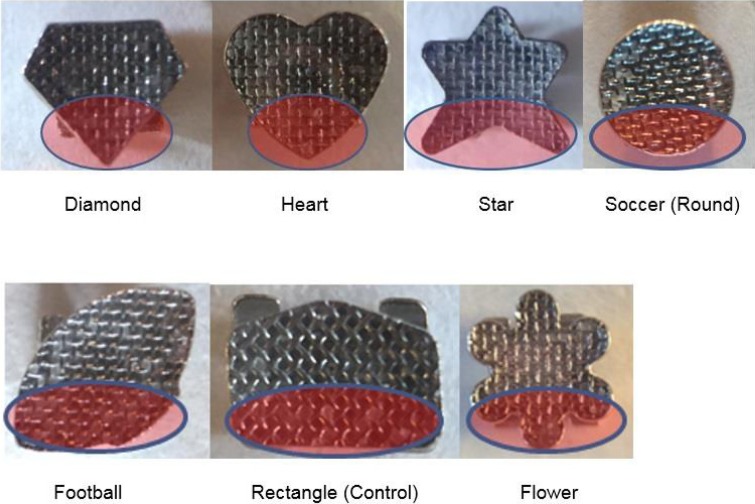
Geometrical Base Shape Comparison between shaped brackets and control group. At the incisal base extension, one point convergence directly over the vertical plane of the bracket base is observed, with more pronounced tip seen with diamond.

Multitude of conclusions can be drawn as a result of intra test group comparison. A statistically significant reduction in SBS (N) was noted for both heart and diamond base shapes when compared against football and flower. A plausible explanation to this two-fold reduction in SBS (N) may lie within its geometrical shape. Visual analysis reveals similar trait observed in both heart and diamond base unseen in all other groups (Fig. 3). At the incisal base extension, one point convergence directly over the vertical plane of the bracket base is observed, with more pronounced tip seen with diamond. Non-homogenous stress distribution within the bracket-adhesive-enamel base during force application has been well-documented using finite element (FE) analysis model (15-17). As with many *in vitro* shear test studies, inciso-gingivally directed force has been shown to concentrate stress at the region of incisal base extension (15-17). Since the edge of the bonded area is most susceptible to stress, crack initiated in this region will likely propagate, resulting in bond failure (17).

It can be interpreted that peak stress concentration at the prominent tip of the diamond base shape may have initiated a crack, resulting in bond failure. This similar process may have occurred at the converging end of heart shaped base also; however, the blunt tip may have provided a better distribution of peak stress resulting in slightly higher failure force. Although, the prominent tip is also observed with the star base shape, presence of two tips at the incisal base extension may have reduced the concentrated peak stress by distributing the force to two separate terminal ends. Similar interpretation can be drawn when comparing soccer (round) versus flower base shape. The presence of three rounded ends in the incisal base extension for flower may have allowed for force distribution to three separate areas in comparison to one rounded end seen with soccer (round) base shape, resulting in the reduction of concentrated peak stress.

Analysis of ARI score revealed inconclusive findings. With diamond and football shape being the exception, bond failure occurred at the bracket-adhesive interface, in alignment with previous findings observed with metal brackets (15). Though football and diamond shape had a similar location of failure, one exhibited the highest mean SBS (N) in contrast to lowest mean SBS (N) observed in the other. Therefore, analysis of ARI score showed no relationship between total applied force and bond failure site, coinciding with previous study (18). The limitations of the study are as follows: Wide scatter of data recorded was observed for all groups in this study. This large standard deviation may be attributed to the culminating effect of multiple factors including base size, adhesive resin thickness, substrate, and applied force location. As previously stated, substantially larger base size used in the study is not typical of a standard clinical bracket. Consequently, the adaptability of the base to the bonding surface is decreased resulting in a myriad of bond strength variating factors (19).

In addition, SBS (N) value recorded may be underestimated due to the substrate used. Indication of bovine incisor as an appropriate substrate for bond strength study has been well documented in the past due to its similarity in histochemical composition to human enamel (13). However, one distinctive difference is the enamel formation rate between bovine and human. Bovine enamel develops much more rapidly during formation than human teeth. At a higher formation rate, inclusion of large crystal grains and lattice defect is more likely, resulting in 21-44 % SBS reduction (20,21). Despite human maxillary central incisor being the ideal substrate for *in vitro* bond strength study, trend towards conservative treatment makes it extremely difficult to obtain large quantities of non-carious, structurally sound tooth (20). The option to use human premolar teeth was not viable since shaped bracket used in this study is only manufactured for maxillary anterior teeth.

Lastly, the location of applied force may have contributed significantly to large range in SBS recorded. Typically, for *in vitro* bond study, shear force is applied at the enamel-resin interface. For this study, shearing force was applied at the ligature groove to maintain consistent location of force for all base shapes. As the distance of applied force from the enamel surface is increased, a moment of force is being introduced (22). As a result, shifting of shear stress to tensile, compressive, and peel stress becomes increasingly large (22). Studies have shown statistically significant difference between shear strength (7.71 MPa) compared to tensile (2.29 MPa) and compressive (2.98 MPa) bond strength (18). Fracture is most likely to occur at the region exhibiting the lowest force carrying capacity, ultimately resulting in bond failure. Klocke *et al.* observed a 49.3% reduction in SBS as well as 25% increase in bracket failure when an applied force was moved from the bracket-resin to the ligature groove (22).

Considerations must be taken into account prior to drawing any conclusion on the clinical performance of shaped brackets. As with any SBS study, it is often difficult to extrapolate *in vitro* data and apply it to clinical situations due to varying testing methods between studies (23). Therefore, direct inference to the clinical performance of these base shapes cannot be made. The often cited, clinically acceptable bond strength of 5.8-7.9 MPa suggested is based on a standard clinical size bracket (7). Since SBS reported in MPa is dependent on the nominal bracket base area, direct comparison of the results obtained may not be suitable. It would be more appropriate to compare total SBS in Newtons. Presently, there is no consensus on the clinically acceptable bond strength provided in regards to total force. However, bracket displacement force of 5-13 kg (49 N-147 N) in the anterior region has been reported (7). Without accounting for the possible underestimated force value observed in this study, rectangle, flower, and football base shapes yielded a mean SBS (N) value strong enough to resist normal masticatory forces.

## Conclusions

In conclusion, based on the data obtained from this study, it can be concluded that bracket base shape has an effect on SBS. When SBS is reported in Newtons, higher bond strength was observed for rectangle, flower, and football shape. These geome-trical shapes may allow for superior force distribution within the enamel-resin-base system when compared to round, star, di-amond and heart. Round and star shape yielded marginally superior bond strength than diamond and heart. Orthodontic bracket with one converging tip over the vertical axial plane within the incisal base has shown to exhibit lower bond strength.
